# The Heart Has Intrinsic Ketogenic Capacity that Mediates NAD^+^ Therapy in HFpEF

**DOI:** 10.1161/CIRCRESAHA.124.325550

**Published:** 2025-04-11

**Authors:** Yen Chin Koay, Bailey McIntosh, Yann Huey Ng, Yang Cao, Xiao Suo Wang, Yanchuang Han, Saki Tomita, Angela Yu Bai, Benjamin Hunter, Ashish Misra, Christopher M. Loughrey, Paul G. Bannon, Sean Lal, Aldons J. Lusis, David M. Kaye, Mark Larance, John F. O’Sullivan

**Affiliations:** 1 Faculty of Medicine and Health, School of Medical Sciences (Y.C.K., B.M., Y.H.N., X.W., Y.H., S.T., A.Y.B., B.H., P.G.B., S.L., M.L., J.F.O.), The University of Sydney, New South Wales, Australia.; 2 Cardiometabolic Medicine (Y.C.K., B.M., Y.H.N., X.W., Y.H., P.G.B., S.L., J.F.O.), The University of Sydney, New South Wales, Australia.; 3 Charles Perkins Centre (Y.C.K., B.M., Y.H.N., X.W., Y.H., B.H., P.G.B., S.L., M.L., J.F.O.), The University of Sydney, New South Wales, Australia.; 4 Precision Cardiovascular Laboratory (B.H., S.L.), The University of Sydney, New South Wales, Australia.; 5 Heart Research Institute (A.M.), The University of Sydney, New South Wales, Australia.; 6 Division of Life Sciences and Medicine, Department of Cardiology, The First Affiliated Hospital of USTC (Y.C.), University of Science and Technology of China (USTC), Hefei.; 7 Division of Life Sciences and Medicine, School of Basic Medical Sciences (Y.C.), University of Science and Technology of China (USTC), Hefei.; 8 School of Cardiovascular and Metabolic Health and School of Biodiversity, One Health and Veterinary Medicine, College of Medical, Veterinary and Life Sciences, University of Glasgow, United Kingdom (C.M.L.).; 9 Department of Cardiothoracic Surgery (P.G.B., J.F.O.), Royal Prince Alfred Hospital, Camperdown, New South Wales, Australia.; 10 Department of Cardiology (S.L., J.F.O.), Royal Prince Alfred Hospital, Camperdown, New South Wales, Australia.; 11 The Baird Institute for Applied Heart and Lung Surgical Research, Sydney, New South Wales, Australia (P.G.B., S.L., J.F.O.).; 12 Department of Medicine, Microbiology and Human Genetics, University of California, Los Angeles (A.J.L.).; 13 Department of Cardiology, Alfred Hospital, Melbourne, Victoria, Australia (D.M.K.).; 14 Heart Failure Group, Baker Heart and Diabetes Institute, Melbourne, Victoria, Australia (D.M.K.).; 15 Faculty of Medicine, Nursing, and Health Sciences, Central Clinical School, Monash University, Melbourne, Victoria, Australia (D.M.K.).; 16 Faculty of Medicine, Technische Universität Dresden, Germany (J.F.O.).

**Keywords:** heart failure, ketone bodies, myocardium, oxygen consumption, stroke volume

## Abstract

**BACKGROUND:**

Heart failure with preserved ejection fraction (HFpEF) has overtaken heart failure with reduced ejection fraction as the leading type of heart failure globally and is marked by high morbidity and mortality rates, yet with only a single approved pharmacotherapy: SGLT2i (sodium-glucose co-transporter 2 inhibitor). A prevailing theory for the mechanism underlying SGLT2i is nutrient deprivation signaling, of which ketogenesis is a hallmark. However, it is unclear whether the canonical ketogenic enzyme, HMGCS2 (3-hydroxy-3-methylglutaryl-coenzyme A synthase 2), plays any cardiac role in HFpEF pathogenesis or therapeutic response.

**METHODS:**

We used human myocardium, human HFpEF and heart failure with reduced ejection fraction transcardiac blood sampling, an established murine model of HFpEF, ex vivo Langendorff perfusion, stable isotope tracing in isolated cardiomyocytes, targeted metabolomics, proteomics, lipidomics, and a novel cardiomyocyte-specific conditional HMGCS2-deficient model that we generated.

**RESULTS:**

We demonstrate, for the first time, the intrinsic capacity of the human heart to produce ketones via HMGCS2. We found that increased acetylation of HMGCS2 led to a decrease in the enzyme’s specific activity. However, this was overcome by an increase in the steady-state levels of protein. Oxidized form of nicotinamide adenine dinucleotide repletion restored HMGCS2 function via deacetylation, increased fatty acid oxidation, and rescued cardiac function in HFpEF. Critically, using a conditional, cardiomyocyte-specific HMGCS2 knockdown murine model, we revealed that the oxidized form of nicotinamide adenine dinucleotide is unable to rescue HFpEF in the absence of cardiomyocyte HMGCS2.

**CONCLUSIONS:**

The canonical ketogenic enzyme, HMGCS2, mediates the therapeutic effects of the oxidized form of nicotinamide adenine dinucleotide repletion in HFpEF by restoring normal lipid metabolism and mitochondrial function.

**Novelty and Significance**

**What Is Known?**

- Heart failure with preserved ejection fraction (HFpEF) is causally related to comorbidities such as obesity, diabetes, and hypertension.
- Oxidized form of nicotinamide adenine dinucleotide repletion therapy, via supplementation with nicotinamide riboside or nicotinamide, reverses cardiac dysfunction associated with HFpEF.

**What New Information Does This Article Contribute?**

- We demonstrate, for the first time, the heart’s intrinsic ability to produce its own ketones.
- The cardiomyocyte ketone production pathway is a critical metabolic contributor to the HFpEF phenotype.
- The ketogenic enzyme, HMGCS2 (3-hydroxy-3-methylglutaryl-coenzyme A synthase 2), plays a critical role in mediating the therapeutic effects of the oxidized form of nicotinamide adenine dinucleotide repletion, mediating the restoration of lipid metabolism and energy production that is essential for the recovery of HFpEF.

This study demonstrates the important role of the HMGCS2 pathway in the pathogenesis and therapeutic management of HFpEF. Using a cardiomyocyte-specific, conditional HMGCS2-deficient murine model, we demonstrated that an oxidized form of nicotinamide adenine dinucleotide repletion therapy is ineffective in treating HFpEF when cardiomyocyte HMGCS2 is absent. This finding indicates that the efficacy of HFpEF treatment via this therapy depends on the HMGCS2 pathway, which is integral to normalizing lipid metabolism and mitochondrial function. This study not only advances our understanding of cardiac ketone metabolism but also reframes our understanding of its role, emphasizing the mechanistic importance of the myocardial ketogenic pathway in HFpEF.

**Meet the First Author, see p 1072 | Editorial, see p 1131**

Of all organs, the heart has the highest energy requirements, resting metabolic rate per unit mass (440 kcal/kg per day),^1^ and the highest oxidative demand.^2^ Therefore, it is critical that the heart can remain flexible in its choice of substrates in the face of changing nutrient supply, metabolic stress, and pathological injury. Certain states, such as diabetes and heart failure, impair this flexibility. In these states, alternative substrates such as ketone bodies become extremely important. It is thought that ketone bodies are a favored substrate under conditions of stress and metabolic inflexibility as they are a thrifty substrate.^3^ Compared with fatty acids (FAs), ketones are more energetically efficient, yielding more energy per unit cost in oxygen consumption (P/O [phosphate/oxygen] ratio).^4–6^ Recently, both clinical^7^ and preclinical^8^ data demonstrated the beneficial effects of ketone administration in heart failure with preserved ejection fraction (HFpEF).^9^ The first, and to-date only, approved class of pharmacotherapy for HFpEF is SGLT2i (sodium-glucose co-transporter 2 inhibitor). SGLT2i is suggested to enhance cardiac metabolism by potentially increasing myocardial ATP production through improved availability of ketone bodies.^10^ The canonical ketogenic enzyme is HMGCS2 (3-hydroxy-3-methylglutaryl-coenzyme A synthase 2), and its activity is dependent on the oxidized form of nicotinamide adenine dinucleotide (NAD^+^)–mediated deacetylation by SIRT3 (sirtuin 3).^11^ However, it is unknown whether HMGCS2 is active in the heart. Two studies published in 2021 showed that supplementation with precursors of NAD^+^ in preclinical models of HFpEF, nicotinamide^12^ or nicotinamide riboside (NR),^13^ activated NAMPT (nicotinamide phosphoribosyltransferase), a key enzyme in the NAD^+^ salvage pathway,^13^ leading to improved diastolic dysfunction in preclinical models of HFpEF.

Mechanistic study of HFpEF has been challenging due to the heterogeneity of clinical presentation, poor diagnostic criteria, and the absence of clinically representative models. Recently, it was shown that invasive hemodynamic assessment during exercise provides superior diagnostic^14^ and predictive^15^ classification of HFpEF, and exercise pulmonary capillary wedge pressure has been used to define inclusion criteria for clinical trials in HFpEF.^16–18^ Furthermore, a robust murine model of HFpEF was recently developed.^19^ Therefore, our goals were to (1) use this murine model to identify mechanisms underlying myocardial metabolic flexibility and the role of myocardial ketone utilization in HFpEF, (2) examine ketone metabolism in human myocardium, (3) determine cardiac ketone uptake in human HFpEF (compared with heart failure with reduced ejection fraction [HFrEF]) where the clinical phenotype is carefully defined using invasive exercise hemodynamics, (4) comprehensively trace metabolic flux through all relevant FA and ketone substrates relevant to ketogenesis, and (5) mechanistically determine the role of ketogenesis in HFpEF and its relationship to the therapeutic effects of NAD^+^ repletion.

## Methods

### Data Availability

Data sets have been made publicly available at the ProteomeXchange Consortium via the PRIDE partner repository with identifiers PXD026582 and PXD033027. Any additional information and data supporting this study are available from the corresponding authors upon reasonable request.

### Animals and Husbandry

All procedures involving animals received ethics approval from the Institutional Animal Ethics Committee at The University of Sydney (protocol 2017/1294; 2021/1885). C57BL/6 wild-type mice (male, 5-week-old; n=20) were obtained from The Australian BioResources Pty Ltd Facility in Moss Vale, New South Wales, Australia. Mice in the control group received a normal chow diet (SF14-162, Specialty Feeds Pty Ltd), and mice in the treatment group were fed a combination of high-fat diet (HFD; 60% calories from lard, SF18-072, Specialty Feeds Pty Ltd) and L-N^G^-nitro arginine methyl ester (L-NAME; 0.5 g/L in drinking water). A separate cohort of C57BL/6 male mice (aged 6–7 weeks; n=18) was fed a chow or HFD (without L-NAME). All mice were euthanized after 5 weeks of different dietary regimens.

In a separate experiment for NR study, 15 mice were randomly divided into 2 groups: the HFpEF group (n=8) and the HFpEF+NR group (n=7). In the HFpEF group, mice were fed an HFD and were given drinking water containing L-NAME (0.5 g/L, pH adjusted to 7.4) for 9 weeks. For NR treatment, mice in this group were initially fed with the same diet and drinking water as the HFpEF group for 5 weeks to develop HFpEF, after which their diet was switched to an HFD incorporated with NR (SF20-100, Specialty Feeds Pty Ltd; NR was purchased from ChromaDex; 400–500-mg/kg body weight per day) and drinking water containing L-NAME for another 4 weeks. Mice were euthanized after 9 weeks of different dietary regimens. Conditions in which animals are maintained are described in the Supplemental Methods.

HMGCS2 floxed mice (Hmgcs2^fl/fl^), created by The Yilmaz Laboratory^20^ and cataloged under MMRRC stock 068091, were crossed with αMHC (α-myosin heavy chain)-CreER (Cre-loxP recombination) mice to produce offspring with a cardiomyocyte-specific deficiency of Hmgcs2 mice. The genotypes of the mice were verified using a polymerase chain reaction. The specific deletion of Hmgcs2 in tissues was confirmed through Western Blot analysis. Mice with cardiomyocyte-specific Hmgcs2-deficiency (αMHC-CreER, Hmgcs2^fl/fl^), along with control groups (αMHC-CreER mice and Hmgcs2^fl/fl^ mice), were subjected to the same 12-week treatment protocols for HFpEF and NR.

### Anesthesia for Mice Echocardiography

Echocardiography was performed in anesthetized mice. Anesthesia was commenced using 2% to 3% isoflurane in oxygen in an induction chamber. Anesthesia was maintained during imaging using 1% to 2% isoflurane in oxygen delivered via nose cone. All animal studies were approved by the Institutional Animal Ethics Committee at The University of Sydney.

### Echocardiography Measurements

Echocardiography was performed 4 weeks after the beginning of the study for the 5-week model and weeks 5 and 9 for the 9-week model. A Vevo2100 system high-resolution ultrasound system with a 40-MHz linear probe (FUJIFILM VisualSonics, Inc, Canada) was used to perform echocardiography on mice 5 weeks after commencement of diets. A detailed description of the echocardiographic parameters is described in the Supplemental Methods.

### Body Composition

Longitudinal measures of body composition were assessed using an EchoMRI 900 (EchoMRI, TX) before culling at each experimental end point.

### Blood Glucose and Insulin Measurement

Oral glucose tolerance tests were performed via oral gavage (2-g/kg lean mass) after 6 hours of fasting. Basal blood glucose was measured through tail vein bleeding using a clinical glucometer (Accu-Chek Performa, Roche Diagnostics Australia Pty Ltd). Blood glucose was measured 15, 30, 45, 60, and 90 minutes after glucose administration. Blood insulin levels were determined by the Ultra Sensitive Mouse Insulin ELISA kit (Crystal Chem Inc). The area under the curve was derived from an oral glucose tolerance test, which indicates the time taken to clear a bolus dose of glucose from the bloodstream and return to basal levels. The homeostasis model assessment of insulin resistance was used as an estimation for global insulin resistance (IR) for each group.^21^

### Human Myocardial Tissue

Left ventricular myocardial samples were obtained from healthy donors and patients with hypertrophic cardiomyopathy from the Sydney Heart Bank, as described in more detail in the Supplemental Methods. Written consent was obtained to use tissue specimens for research. The study was approved by the Ethics Committee of The University of Sydney (USYD 2016/923, 2020/ETH01161, and USYD 2021/122) and was conducted in accordance with the Declaration of Helsinki.

### Human Tissue Microarray and Immunohistochemistry

The cryopreserved samples (healthy donor [n=11; mean age, 44 years; 50% male] and hypertrophic cardiomyopathy [n=8; mean age, 53 years; 54.55% male]) that were used for the tissue microarrays were thawed and fixed in ice-cold 10% neutral buffered formalin and then embedded in paraffin. Tissue microarray myocardial sections were obtained and underwent immunohistochemistry, using previously described methods.^22^ Detailed histochemistry and quantitative analysis of immunostaining and histochemical staining are described in the Supplemental Methods.

### Invasive Exercise Hemodynamics and Transcardiac Gradients

Patients with HFpEF (n=22) were referred to the Department of Cardiology, Alfred Hospital, for investigation of symptoms consistent with a diagnosis of heart failure (New York Heart Association [NYHA] II-III) in the presence of a left ventricular ejection fraction >50%, with HFrEF cohort (n=20) having left ventricular ejection fraction (<40%). Table S1 shows the characteristics of patients with HFpEF and HFrEF. Exclusion criteria included significant coronary artery disease, which had not been revascularized; moderate or greater aortic or mitral valve disease; infiltrative, restrictive, or hypertrophic myocardial disease; pericardial constriction; or significant right ventricular disease. The diagnosis of HFpEF was confirmed by the presence of an end-expiratory pulmonary capillary wedge pressure ≥15 mm Hg at rest or ≥25 mm Hg during symptom-limited exercise. The study was approved by the institutional ethics review committee, and all participants provided written informed consent.

### Proteomics

The sample preparation and proteomics workflows were performed based on previously published methods.^23^ Using a Thermo Fisher Dionex Ultimate 3000 UHPLC, peptides in 5% (vol/vol) formic acid (500 ng) were directly injected onto a 50 cm×75 µm fused-silica analytical column with a ≈10-µm pulled tip containing C18 material (Dr Maisch, Ammerbuch, Germany, 1.9 µm, 130A), coupled online to a nanospray ESI (electrospray ionization) source. Peptides were resolved over a gradient of 5% to 40% acetonitrile over 120 minutes with a flow rate of 300 nL/min. Peptides were ionized by electrospray ionization at 2.3 kV. Tandem mass spectrometry analysis was performed on a Q-Exactive HF-X mass spectrometer using HCD (higher energy collisional dissociation) fragmentation (Thermo). Additional information on the sample preparation and proteomics workflows was provided in the Supplemental Methods.

### Sample Preparation for Metabolomics and Lipidomics

Briefly, ≈30 to 50 mg of powdered mice heart tissues were subjected to metabolite and lipid extraction before liquid chromatography-tandem mass spectrometry analysis. A stepwise extraction method was performed as previously described, using methanol/chloroform/water to efficiently extract lipids and metabolites from the tissues.^24^

### Targeted Metabolomics

For targeted metabolomic analysis, a liquid chromatography-tandem mass spectrometry system composed of a Shimadzu Nexera LC-30AD UHPLC (Shimadzu Corporation, Kyoto, Japan) system coupled to a QTRAP6500 mass spectrometer (AB Sciex, Foster City, CA) was used to measure hydrophilic metabolites in positive and negative ionization modes. Targeted metabolite profiling used in this study was established using reference standards for each individual metabolite to determine mass spectrometer (MS) multiple reaction-monitoring transitions, declustering potentials, collision energies, and chromatographic retention time, as described previously.^25^

The analysis software Sciex OS 2.0 (ABSciex) was used for MRM Q1/Q3 peak integration of the raw data files (Analyst software, version 1.6.2; ABSciex). The peak area corresponds to the abundance of that metabolite; the abundance values were then normalized to their bookended pooled tissue extracts in the subsequent analysis, which were included after every 4 study samples in the sample queue, to account for temporal drift in instrument performance.

### Lipidomics

For analysis of lipid extracts from mice hearts, isotopically labeled lipid standard mixture (SPLASH Lipidomix Mass Spec Standard, Avanti Polar Lipids) was spiked into each sample, and the concentrations of identified lipid species from 14 major lipid classes included in the SPLASH were calculated relative to the known concentration of isotopically labeled internal standards included in the SPLASH standard mixture.

Lipidomic profiling in this study used a Thermo Scientific Vanquish UHPLC system and Thermo Scientific Q-Exactive HF-X Hybrid Quadrupole-Orbitrap mass spectrometer, with lipid separation performed on a Thermo Scientific Acclaim C30 column (2.1×150 mm, 2.6 µm), operated at 45 °C, and a flow rate of 260 µL/min. Lipid extracts from the mice hearts were analyzed using the LC conditions and MS instrument conditions in both positive and negative ion modes, as described previously.^26,27^ Lipid Search 4.1 software was used to align and process each acquired raw file’s search results and identify the lipid species present in the heart extracts.^28^

### Tissue Homogenization and Lysis for Enzymatic Activity Assay

For the determination of HMGCS2 activity in the 5-week mice model, a total of 2 to 3 mouse heart tissues were pooled together to ensure sufficient material for the protein extraction for each assay (n=3 per group for control and n=4 per group for HFpEF). For the 9-week mice model, a total of 2 to 3 mouse heart tissues were combined and pooled together for each assay (n=3 per group for HFpEF and n=3 per group for NR-treated mice). Heart extracts were prepared by lysis in 500 μL of ice-cold mammalian cell lysis extraction mixture containing protease inhibitor cocktail solutions (MCL1 [myeloid cell leukemia‐1]; Sigma-Aldrich). Tissues were homogenized by Qiagen TissueLyser LT (50 cycle/s) for 2 minutes. Particulate matter was then separated by centrifugation for 10 minutes at 4 °C at 11 000*g*. The resulting clear supernatant was collected for incubation. Human myocardial tissues were subjected to the same tissue homogenization procedures for extract preparation for mice.

### HMGCS2 Enzymatic Activity

The protein content of the lysate mixture was determined using the bicinchoninic acid protein assay kit (Pierce Biotechnology, Rockford, IL) according to the manufacturer’s instructions. HMGCS2 activity assay in mouse and human heart tissue was performed with a modification of previously published protocols.^29^ Incorporation of isotope-labeled [1,2-^13^C_2_]Ac-CoA (acetyl-coenzyme A) to isotope-labeled HMG-CoA (β-hydroxy β-methylglutaryl-coenzyme A) and acetoacetate at pH 8 and temperature set to 37 °C was measured. Briefly, 250 μL of reaction mixture containing 50-mmol/L trisaminomethane, 0.01 mmol/L of acetoacetyl-CoA (coenzyme A), 1 mmol/L of [1,2-^13^C_2_]Ac-CoA (Sigma, 658650-5MG), and 150 μL (0.225 mg/mL) of the heart tissue homogenate was added for 5-week model, or 150 μL (0.100 mg/mL due to lower protein content) of the heart tissue homogenate was added for 9-week model. The reaction proceeded at 37 °C for 5, 10, 20, 30, 45, 60, 90, 120, 150, 180, and 210 minutes, with the removal of 30 μL of aliquots of the reaction mixture, and the enzymatic reaction was stopped immediately by adding 5 μL of 5-M perchloric acid starting at time=0 and the indicated time points. Measurement of ^13^C-labeling in substrate (Ac-CoA), intermediates (acetoacetyl-CoA and HMG-CoA), and product of the ketogenic pathway (acetoacetate) were performed using QTRAP6500 mass spectrometer (AB Sciex, Foster City, CA) coupled to a Shimadzu Prominence LC system. Concentration values of ^13^C_4_-acetoacetate and ^13^C_6_-HMG-CoA were obtained in the unit of µmol/L. These values were divided by the incubation time (hours) and the protein concentrations, respectively, in the unit mg/L. The resulting enzymatic activity values were expressed in total ^13^C_4_-acetoacetate and ^13^C_6_-HMG-CoA generated µmol/mg protein per hour, which were transformed to the final unit nmol·mg/p per hour for ^13^C_4_-acetoacetate. Additional details on the metabolite extraction and measurement of enzymatic activity were provided in the Supplemental Methods.

### Western Blot and Immunoprecipitation Assay

The protein concentration of the resultant supernatant from the enzymatic assay was determined using the bicinchoninic acid protein assay kit (Pierce), following the manufacturer’s instructions. Proteins were separated by 4% to 15% Mini-PROTEAN TGX Precast Gel (Bio-Rad) and transferred to a PVDF membrane (Thermo Fisher Scientific). The blots were then visualized using the ImageQuant LAS 4010 digital imaging system (GE Healthcare). For the immunoprecipitation assay, protein concentrations of the tissue lysates from the enzymatic activity assays were determined using the bicinchoninic acid protein assay kit (Pierce, Rockford); 150 μg of the protein lysates were incubated with anti-acetyllysine or anti-HMGCS2 overnight at 4 °C, followed by incubation with 25-µL Protein G Sepharose Beads (2 mg/mL; GE Healthcare) for 2 hours at 4 °C. The beads were washed 4× with immunoprecipitation assay buffer before analysis. Proteins were detected using the following primary antibodies: Abcam-HMGCS2 (HMGCS2; ab137043), β-actin (ab8226), and cell signaling-acetylated lysine (9814S).

### Statistical Analysis

Normalized and absolute values of fold changes among groups were calculated for metabolomic and proteomic data and were Log2 transformed. The normality and the homogeneity of variance of the data were tested. Comparisons between 2 groups were performed using the Student *t* test or the Mann-Whitney *U* test, depending on the distribution of the data. We assessed data normality through the Shapiro-Wilk test (setting α at 0.05) and by reviewing normal Q-Q plots to confirm the absence of significant deviations from a normal distribution. For the comparison of multiple groups, 2-way ANOVA followed by the Tukey post hoc multiple comparisons test was used. Transcardiac ketone body gradients were compared using the Welch 2-Sample *t* test, using R, version 4.0.2. Protein outputs were corrected for multiple tests using the Benjamini-Hochberg correction, with significance being set at *P*≤0.05 at an false discovery rate (FDR) of 5% and plotted using Tableau 2023.1 or GraphPad Prism software 10.1.2. Of note, *P* values for other analyses reported in this study have not been adjusted for multiple comparisons, and each analysis was treated independently.

## Results

### HFpEF Hearts Have Suppressed Glycolysis and Upregulated PDK4 Causing Metabolic Inflexibility

**Figure 1.**
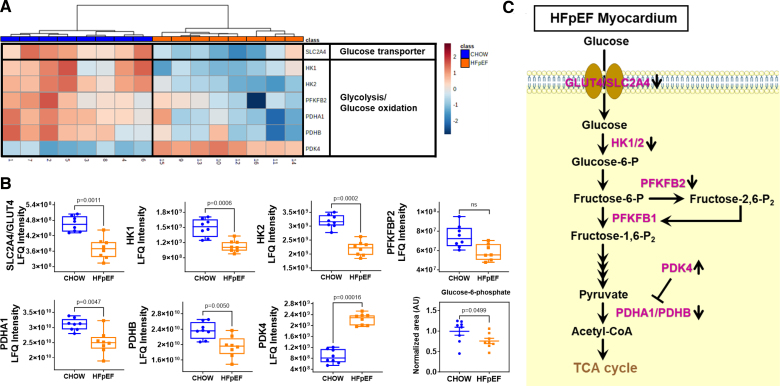
**Myocardial glycolytic metabolism. A**, Heatmap showing decreased expression of glucose transporter and glycolytic enzymes in heart failure with preserved ejection fraction (HFpEF) myocardium. Protein label-free quantitation (LFQ) intensities were normalized and visualized using MetaboAnalyst 5.0. Each column in the figure represents data from an individual mouse. A color scale on the upper right side of the figure indicates normalized protein abundance (ranging from 2.0 to −2.0). **B**, Box and whisker plots with individual dot points in heart tissue from HFpEF (n=8) vs chow (n=8) illustrating significant reductions of several rate-limiting enzymes critical for the regulation of glycolysis and glucose oxidation, with upregulation of PDK4 (pyruvate dehydrogenase kinase 4) that inhibits the pyruvate dehydrogenase complex leading to metabolic inflexibility. *P*<0.05 is statistically significant, and exact values are specified in corresponding figures. Data were analyzed by the Mann-Whitney *U* test. **C**, Schematic summarizing the glycolytic pathway changes. GLUT4 indicates glucose transporter type 4; HK1, hexokinase 1; HK2, hexokinase 2; PDHA1, pyruvate dehydrogenase-A1; PDHB, pyruvate dehydrogenase B; PFKFB2, 6-phosphofructo-2-kinase/fructose-2,6-bisphosphatase 2; SLC2A4, solute carrier family 2, facilitated glucose transporter member 4; and TCA, tricarboxylic acid.

Compared with mice fed chow, mice fed a combination of HFD and L-NAME for 5 weeks (HFpEF mice) showed significantly increased fat mass compared with chow-fed mice along with impaired glucose tolerance and insulin resistance as indicated by the measurement of glucose area under the curve and homeostatic model assessment of insulin resistance (Figure S1A through S1C). HFpEF mice displayed cardiac hypertrophy as indicated by increased heart-weight-to-tibial-length ratio (Figure S1D), elevated LV filling pressures by transmitral to tissue doppler ratio (Figure S1E and S1F), impaired global longitudinal strain (Figure S1G), and pulmonary congestion by increased wet/dry lung weight ratio (Figure S1H). The murine HFpEF myocardial proteome displayed decreased levels of glucose transporters along with depleted glycolytic and glucose oxidative substrates consistent with an insulin-resistant heart (Figure 1A). There were decreased protein abundances of several rate-limiting enzymes critical for the regulation of glucose uptake, glucose phosphorylation, and the conversion of pyruvate to Ac-CoA (Figure 1B and 1C). Levels of the insulin-responsive glucose transporter that regulates glucose entry in the heart, GLUT4 (glucose transporter type 4; also known as SLC2A4 [solute carrier family 2, facilitated glucose transporter member 4^30^; fold change (FC)=0.80; *P*=0.0011] and downstream HK [hexokinase] 1 and 2 [HK1: FC=0.75; *P*=0.0006; HK2: FC=0.69; *P*=0.0002]), were significantly decreased in HFpEF myocardium (Figure 1B). No change was detected in the PFKFB2 (6-phosphofructo-2-kinase/fructose-2,6-bisphosphatase 2) protein levels. HFpEF hearts had over 2.5-fold increase in PDK4 (pyruvate dehydrogenase kinase 4) expression (FC=2.60; *P*=0.00016), which can reduce glucose oxidation by deactivating the PDHC (pyruvate dehydrogenase complex), a key mitochondrial enzyme that catalyzes the oxidative decarboxylation of pyruvate to Ac-CoA, linking glycolysis to the tricarboxylic acid (TCA) cycle.^31^ Two subunits of pyruvate dehydrogenase (E1), PDHA1 (pyruvate dehydrogenase-A1 LV) and PDHB (pyruvate dehydrogenase B), were significantly decreased (PDHA1: *P*=0.0047; PDHB: FC=0.83; *P*=0.0050) in HFpEF myocardium. Glucose-6-phosphate (an important intermediate of glucose metabolism) was significantly decreased (FC=0.71; *P*=0.0499; Figure 1B) in the hearts of HFpEF mice, consistent with the reduction of glucose transport into these hearts (Figure 1C).

### Altered Mitochondrial FAO, Ketogenesis, and Ketone Oxidation in HFpEF Myocardium

**Figure 2.**
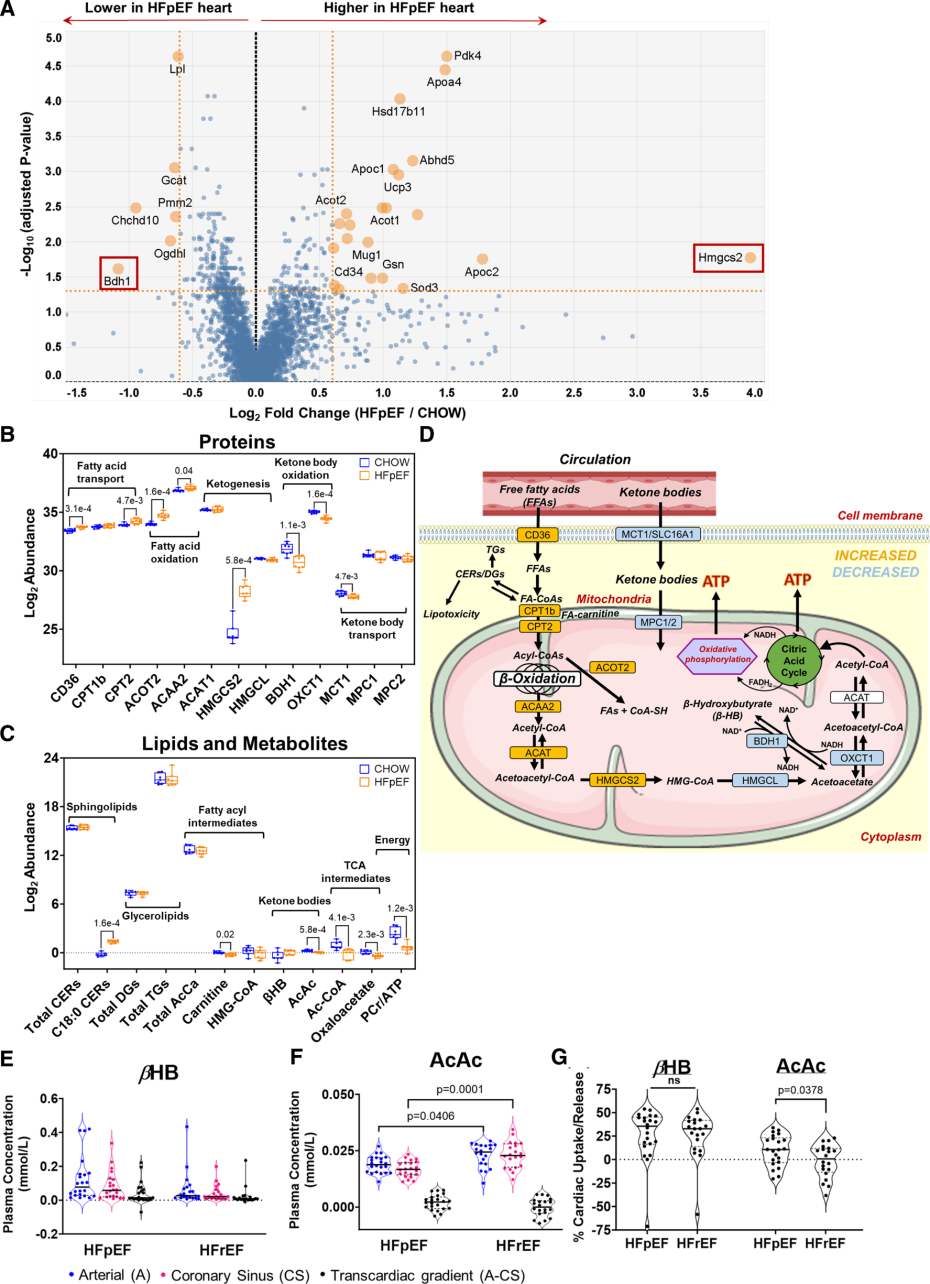
**Proteomics pinpoints perturbed *Hmgcs2* metabolism. A**, Volcano plot depicting differentially abundant proteins in heart failure with preserved ejection fraction (HFpEF) vs control myocardium. The *x* axis displays the log_2_ fold change, and the *y* axis shows the −log_10_ of *P* values derived from a *t* test, adjusted using the Benjamini-Hochberg procedure. The dashed horizontal line shows the cutoff *P* value (<0.05), and the 2 vertical dashed lines represent the fold change cutoff of 1.5 (≈0.6 on log_2_ scale). Box plots illustrating changes in (**B**) proteins involved in fatty acid transport, ketogenesis, and ketone oxidation (n=8/group) and (**C**) substrates and products of the fatty acid transport, fatty acid oxidation, ketogenic, and ketone oxidation pathways in HFpEF group vs control myocardium (n=7/group). The *y* axis represents the log2-scaled abundance of each protein/metabolite. Statistical significance was assessed using the Mann-Whitney *U* test (**B** and **C**). **D**, Schematic summarizing the changes in ketogenic and lipid oxidation pathways with subcellular location indicated. HMGCS2 (3-hydroxy-3-methylglutaryl-coenzyme A synthase 2) is seen as an inflection point between increases and decreases in pathway intermediates; proteins indicated in yellow denote upregulation and blue downregulation in the HFpEF heart. Assessment of myocardial ketones using violin plots illustrated unchanged arterial and coronary sinus concentrations of (**E**) β-hydroxybutyrate (βHB), but lower arterial and coronary sinus concentrations of (**F**) acetoacetate (AcAc) in HFpEF subjects compared with HFrEF subjects. **G**, The percentage of myocardial extraction of ketones illustrated an increase in cardiac uptake of AcAc in HFpEF compared with heart failure with reduced ejection fraction (HFrEF) subjects (human transcardiac gradients: n=22 patients with HFpEF, and n=20 patients with HFrEF). The median of the violin plots is represented by a solid line, while the first and third quartiles are indicated by dashed lines. *P*<0.05 is statistically significant, and precise values are specified in corresponding figures. Data were analyzed by Kruskal-Wallis with the Dunn test (**E**), 2-way ANOVA followed by the Sidak test (**F**), or the Student *t* test (**G**). The percentage of myocardial extraction of ketones was calculated as arterial minus coronary sinus concentration divided by arterial concentrations multiplied by 100. A positive value indicates net uptake by the heart, whereas a negative value indicates net release. ACAA2 indicates acetyl-coenzyme A acyltransferase 2; ACAT1, acetyl-coenzyme A acetyltransferase 1; ACOT2, acyl-coenzyme A thioesterase 2; BDH1, beta-hydroxybutyrate dehydrogenase 1; CD36, cluster of differentiation 36; CER, ceramides; CPT1b, carnitine palmitoyltransferase I; CPT2, carnitine palmitoyltransferase 2; HMGCL, 3-hydroxymethyl-3-methylglutaryl-coenzyme A lyase; MCT1, monocarboxylate transporter 1; MPC, mitochondrial pyruvate carrier; OXCT1, 3-oxoacid coenzyme A-transferase 1; TCA, tricarboxylic acid; and βHB, β-hydroxybutyrate.

As illustrated in the volcano plot in Figure 2A, mitochondrial HMGCS2 changed far more than any other protein, with a 16-fold upregulation in HFpEF myocardium (Figure S2A). HFpEF mice had significantly reduced levels of the myocardial ketone MCT (monocarboxylate transporter) 1 (also called SLC16A1; FC=0.80; *P*=0.0047; Figure 2B; Figure S2A) compared with control mice. Protein abundance of MPC (mitochondrial pyruvate carrier), which is used to transport ketone bodies into mitochondria, was found to be lower in HFpEF hearts. Despite decreased MPC protein levels in HFpEF hearts, there were significantly elevated levels of proteins that facilitate the uptake of FAs into the heart including FA translocase (CD36 [cluster of differentiation 36]; FC=1.2; *P*=3.1×10^−^^4^; Figure 2B; Figure S2A) and FA transporters CPT1b (carnitine palmitoyltransferase I) and CPT2 (carnitine palmitoyltransferase 2; CPT1b: FC=1.1, not significant; and CPT2, FC=1.3; *P*=4.7×10^−3^; Figure 2B; Figure S2A). ACADs (acyl-CoA dehydrogenases) catalyze the first step in mitochondrial β-oxidation of FAs and are classified according to substrate chain-length specificity for FA oxidation (FAO): SCAD (short-chain acyl-CoA dehydrogenase; C4–C6), MCAD (medium-chain acyl-CoA dehydrogenase; C4–C12), long-chain acyl-CoA dehydrogenase (LCAD; C8–C20), and very-long-chain acyl-CoA dehydrogenase (VLCAD; C12–C24). We found that the protein abundance of VLCAD was significantly higher in HFpEF hearts (Figure S2B).

In addition to elevated CPT1b, CPT2 and ACADs, 2 key mitochondrial FAO enzymes, ACOT2 (acyl-CoA thioesterase 2; FC=1.6, *P*=1.6×10^−^^4^; Figure 2B; Figure S2A) and ACAA2 (Ac-CoA acyltransferase 2; FC=1.1; *P*=0.04; Figure 2B; Figure S2A), were also significantly increased. L-carnitine, used for the transport of long-chain FAs into mitochondria for β-oxidation and consumed during FA import for oxidation,^32^ was significantly reduced (FC=0.86; *P*=2.1×10^−^^2^; Figure 2C) in HFpEF myocardium. The HFpEF heart did have increased protein levels of FAO enzymes; critically, however, the activity of FAO was significantly lower (Figure S2C), supported by a significant increase in (accumulation of) medium- and long-chain acylcarnitine concentrations (Figure S2D).

Characteristic of a rate-limiting enzyme, HMGCS2 served as a transition point, whereby upstream enzymes were increased and downstream enzymes were unchanged or decreased (Figure 2B and 2D). Levels of the next enzyme in the cascade, HMG-CoA lyase (HMGCL [3-hydroxymethyl-3-methylglutaryl-coenzyme A lyase]), which catalyzes the cleavage of HMG-CoA to liberate Ac-CoA and acetoacetate, did not change significantly in HFpEF myocardium. Further downstream, mitochondrial matrix enzyme OXCT1 (3-oxoacid CoA-transferase 1; FC=0.66; *P*=1.6×10^−^^4^; Figure 2B; Figure S2A) and BDH1 (beta-hydroxybutyrate dehydrogenase 1; FC=0.47; *P*=1.1×10^−^^3^; Figure 2B; Figure S2A), each involved in ketone body utilization, were both significantly decreased. BDH1 (interconverts βHB [beta-hydroxybutyrate] and acetoacetate) had the greatest fold reduction in murine HFpEF myocardium of all proteins. Cardiac levels of ketone body acetoacetate (acetoacetate; FC=0.88; *P*=5.8×10^−^^4^; Figure 2C), but not βHB, were significantly lower in HFpEF mice. Ac-CoA, a sensitive indicator of decreased energetics, was found to be significantly reduced by ≈2-fold (*P*=4.1×10^−^^3^; Figure 2C) in HFpEF myocardium. HFpEF hearts had a significant reduction in myocardial phosphocreatine-to-ATP ratio (FC=−2.93; *P*=1.2×10^−^^3^; Figure 2C), suggesting an energy-deficient state.^33,34^

Interrogating the contribution of HFD alone (without L-NAME), which provides ketogenic substrates but does not lead to HFpEF, we used HFD only compared with chow in a separate experiment. HFDs alone did not significantly change myocardial HMGCS2, HMGCL, BDH1, or OXCT1 levels (Figure S3A). We also observed no change in the βHB-to-acetoacetate ratio of the HFD hearts compared with control mice hearts (Figure S3B); in a separate comparative experiment, HFpEF mouse myocardium showed a significant increase in βHB-to-acetoacetate ratio (Figure S3C).

### Transcardiac Gradients in Human HFpEF Versus HFrEF Reveal a Predilection for AcAC Uptake Compared With βHB

**Figure 3.**
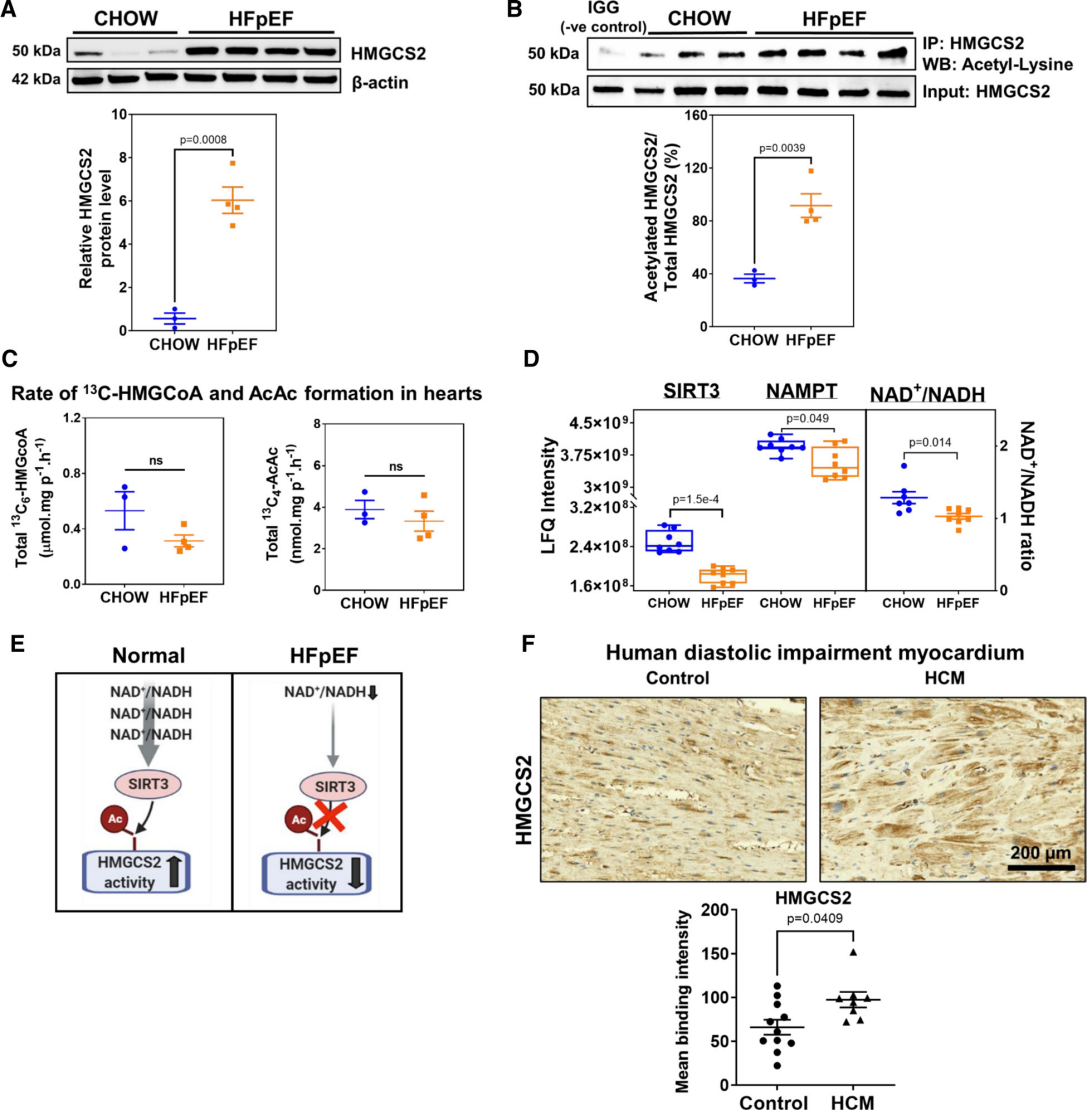
**Oxidized form of nicotinamide adenine dinucleotide (NAD^+^) depletion and reduced myocardial ketogenic specific activity. A**, Representative image of and Western Blot analysis of HMGCS2 (3-hydroxy-3-methylglutaryl-coenzyme A synthase 2) in chow and heart failure with preserved ejection fraction (HFpEF) groups (n=3 mice for chow; n=4 mice for HFpEF). **B**, Immunoprecipitated HMGCS2 from HFpEF myocardium revealed greater acetylation (n=3 mice for chow; n=4 mice for HFpEF). **C**, Total ^13^C_6_-labeled HMG-CoA (β-hydroxy β-methylglutaryl-coenzyme A) and ^13^C_4_-labeled acetoacetate from labeled [1,2-^13^C_2_]-acetyl-CoA was not significantly different in HFpEF vs control myocardium in mice (n=3 mice for chow; n=4 mice for HFpEF). **D**, The bar graph shows the abundance of SIRT3 (sirtuin 3) protein and ratios of NAD^+^/reduced form of nicotinamide adenine dinucleotide (NADH) in hearts from HFpEF mice over controls (n=8 mice/group). **E**, Schematic proposing depletion of NAD^+^ causing decreased SIRT3 that impairs deacetylation, potentially leading to decreased HMGCS2 activity. **F**, Upregulation of HMGCS2 in hypertrophic cardiomyopathy (HCM) samples. Representative immunohistochemistry (IHC) staining for HMGCS2 in the human myocardium (scale bar, 200 µmol/L). Semiquantitative analysis of the mean binding intensity of IHC staining. n=11 for control (healthy donors) and n=8 for HCM. Data represent the mean±SEM. Statistical significance was determined using the Mann-Whitney *U* test (**A**–**D** and **F**), with *P*<0.05 considered significant. NAMPT indicates nicotinamide phosphoribosyltransferase.

In a cohort of patients with HFpEF and patients with HFrEF (Table S1), we determined transcardiac ketone body gradients. All patients underwent invasive hemodynamic assessment before and during exercise. The arterial and coronary sinus βHB levels were not significantly different in patients with HFpEF compared with patients with HFrEF (Figure 2E), while acetoacetate levels were significantly higher in patients with HFrEF compared with patients with HFpEF (Figure 2F). While absolute transcoronary concentration gradient for both βHB and acetoacetate did not differ significantly between the 2 groups (Figure 2E and 2F), the percentage myocardial uptake of acetoacetate (arterial-coronary sinus concentration/arterial concentration*100), but not βHB, was significantly increased in patients with HFpEF compared with patients with HFrEF, suggesting increased consumption of acetoacetate in human HFpEF hearts, consistent with that seen in the HFpEF murine model (Figure 2C). The relative depletion of acetoacetate in murine HFpEF hearts (Figure 3C) is consistent with the increased acetoacetate uptake/utilization compared with βHB in human HFpEF hearts.

### Human Hearts Have Intrinsic Ketogenic Capacity

As described above, comprehensive proteomic profiling revealed that HMGCS2, the canonical rate-limiting ketogenic enzyme, was ≈16-fold (1500%) elevated in HFpEF myocardium (Figure S2A). We wanted to establish definitively whether the heart is capable of synthesizing ketone bodies via this canonical enzyme, which we assessed by measurement of irreversible conversion of [1,2,3,4-^13^C_4_]acetoacetyl-CoA to [1,2,3,4,5,6-^13^C_6_]β-hydroxy β-methylglutaryl-CoA (HMG-CoA) catalyzed by HMGCS2, which is cleaved by HMG-CoA lyase (HMGCL) to generate [1,2,3,4-^13^C_4_]acetoacetate (Figure S4A and S4B). As illustrated in Figure S4A and S4B, in healthy human myocardium, HMGCS2-catalyzed ketogenesis is indeed an active process, with a time-dependent ^13^C enrichment of products HMG-CoA and acetoacetate. The dynamic ^13^C-labeling of HMG-CoA distinguishes this process from reverse thiolase or SCOT (succinyl-coA:3-ketoacid CoA transferase) activity as the source of ^13^C_4_-acetoacetate enrichment.

### HMGCS2-Specific Activity Is Reduced in HFpEF Myocardium but Compensated by Increased Protein Levels

Western blotting confirmed (Figure 3A) the dramatic upregulation of HMGCS2 protein levels initially determined by proteomics (Figure 2A). Immunoprecipitation followed by Western blotting revealed increased acetylation of lysine residues on HMGCS2 (Figure 3B). Despite the elevated acetylation of HMGCS2, the overall activity of the ketogenic pathway, indicated by the rate of formation of [1,2,3,4,5,6-^13^C_6_]HMG-CoA and [1,2,3,4-^13^C_4_]acetoacetate, did not significantly change between the HFpEF and control groups (Figure 3C). While acetylation reduces the enzymatic activity of HMGCS2, the substantial increase in total HMGCS2 protein levels in HFpEF hearts likely compensates for this reduction, maintaining the overall activity of the ketogenic pathway at levels comparable to controls. This was accompanied by decreased levels of SIRT3 (FC=0.76; *P*=1.5×10^−^^4^) in HFpEF myocardium (Figure 3D), a deacetylase that typically activates HMGCS2 through deacetylation. As shown previously in the liver, under normal conditions, sufficient levels of SIRT3 are required to deacetylate HMGCS2 to maintain its active site in the correct conformation for binding substrate acetoacetyl-CoA, that is, maintaining its ketogenic activity.^11^ SIRT3 is dependent on preserved NAD^+^/reduced form of nicotinamide adenine dinucleotide (NADH) ratio,^35^ which we found to be significantly decreased (Figure 3D). Therefore, our results suggest that depleted NAD^+^/NADH leads to reduced SIRT3 activity, resulting in overacetylated HMGCS2 that is dysfunctional (Figure 3D and 3E). In conjunction with the metabolic inflexibility reported above, this served as a stimulus to upregulate HMGCS2 protein levels in the HFpEF myocardium.

Hypertrophic cardiomyopathy shares the diastolic impairment seen in HFpEF but not necessarily the metabolic duress. Nonetheless, in hypertrophic left ventricle (LV) myocardium from individuals without coexisting diabetes, we found upregulation of HMGCS2 staining compared with age and sex-matched healthy donor hearts (Figure 3F).

### Supplementation of NR, a Direct Activator of NAD^+^ Biosynthesis, Activates HMGCS2 and Facilitates Ketone Body Utilization

**Figure 4.**
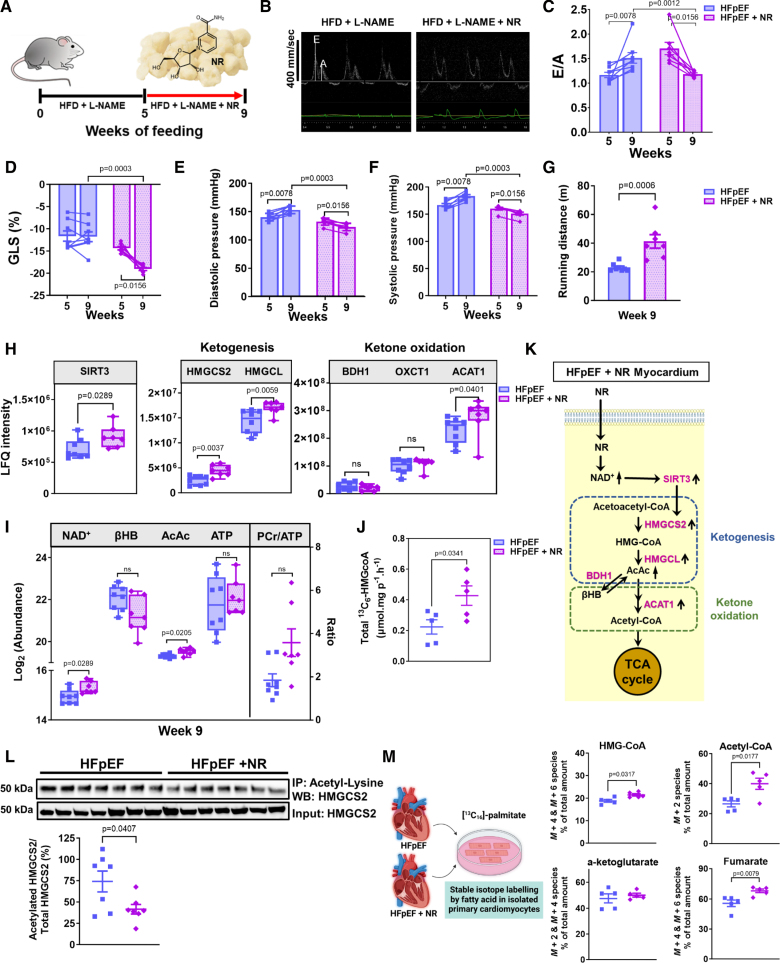
**Dietary supplementation of nicotinamide riboside (NR) significantly improves diastolic dysfunction and exercise tolerance and increases cardiac ketogenesis and ketone oxidation in heart failure with preserved ejection fraction (HFpEF) mice. A**, Schematic overview of NR, oxidized form of nicotinamide adenine dinucleotide (NAD^+^) precursor, and NR feeding protocol to HFpEF mice. **B**, Representative images of mitral pulsed Doppler echocardiography, showing ratio of peak velocity of mitral blood inflow in early diastole (**E**) to peak velocity of mitral blood inflow in late diastole (**A**); (**C**) ratio of peak velocity of mitral blood inflow in early diastole to peak velocity of mitral blood inflow in late diastole (E/A); (**D**) global longitudinal strain (GLS); (**E**) diastolic blood pressure (DBP) and (**F**) systolic blood pressure (SBP); and (**G**) exercise capacity as indicated by running distance in HFpEF (n=8) and NR-treated mice (n=7) at weeks 5 and 9. Data are presented as mean±SEM. Statistical significance was determined using the Wilcoxon matched-pair signed-rank test for paired comparisons between weeks 5 and 9 and the Mann-Whitney *U* test for unpaired comparisons between HFpEF and HFpEF+NR groups at each time point. Cardiac proteome profiling demonstrated increased expression of proteins involved in (**H**) deacetylation, ketogenesis, and ketone oxidation pathway (n=8 for HFpEF and n=7 for HFpEF+NR). **I**, Myocardial metabolite profile and (**J**) total ^13^C_6_-labeled HMG-CoA (β-hydroxy β-methylglutaryl-coenzyme A) from enzymatic activity assay in HFpEF (n=5) vs NR-treated (n=5) myocardium at week 9. Each data point was obtained from 2 to 3 pooled HFpEF and NR-supplemented mouse hearts. **K**, Overall schematic diagram highlighting the changes of cardiac ketogenesis and ketone oxidation in the post-NR therapy mice. **L**, Heart lysates from HFpEF (n=7) and NR-treated (n=7) mice were subjected to immunoprecipitation using an anti-acetyl-lysine antibody and analyzed using anti-HMGCS2 (3-hydroxy-3-methylglutaryl-coenzyme A synthase 2) antibody. **M**, The abundance percentages of isotopologues M+4 and M+6 of HMG-CoA, as well as M+2 isotopologues of acetyl-CoA (coenzyme A), and M+2 and M+4 isotopologues of alpha-ketoglutarate and fumarate, were determined from the metabolism of ^13^C_16_-labeled palmitate (n=5 mice in HFpEF and HFpEF+NR groups). Data represent the mean±SEM. In **H** through **J**, **L**, and **M**, statistical significance was assessed using the Mann-Whitney *U* test. *P*<0.05 is considered significant; exact values are provided in the corresponding figures. HFD indicates high-fat diet; L-NAME, L-N^G^-nitro arginine methyl ester; and ns indicates no significant.

Supplementation with NR, the NAD^+^ precursor, for 4 weeks at 500- to 600-mg/kg body weight per day (Figure 4A) resulted in significantly improved diastolic function (ratio of peak velocity of mitral blood inflow in early diastole to peak velocity of mitral blood inflow in late diastole and global longitudinal strain; Figure 4B through 4D), diastolic blood pressure and systolic blood pressure (Figure 4E and 4F), and exercise tolerance (Figure 4G) compared with the pretreatment group at week 5 and the nontreated HFpEF group at week 9. Notably, replenishing myocardial NAD^+^ levels through NR supplementation significantly increased mitochondrial SIRT3 expression levels (Figure 4H). NR-supplemented HFpEF hearts displayed significantly increased expression levels of HMGCS2 (Figure 4H), as well as HMGCL (Figure 4H), which also depends on SIRT3-mediated deacetylation and is the next enzyme in the ketogenic cascade after HMGCS2. Further downstream, the ketolytic enzyme ACAT1 (Ac-CoA acetyltransferase 1) was significantly increased (Figure 4H), consistent with increased ketone flux and oxidation. Although enzyme BDH1 (Figure 4H) is dependent on NAD^+^/NADH and SIRT3, protein levels did not change with NAD^+^ repletion. NR treatment also effectively elevated myocardial levels of NAD^+^ and ketone body, acetoacetate (Figure 4I), compared with the nontreated HFpEF group. Nevertheless, there was a nonsignificant (*P*=0.07) increase in phosphocreatine/ATP ratio in the NR-treated hearts (Figure 4I), which may suggest a partial improvement in energy homeostasis. This could be due to a continuous utilization of ATP over time, rather than its accumulation, potentially driven by enhanced ketone generation and utilization as an alternative cardiac energy substrate. There was also an increased rate of formation of ^13^C_6_-labeled HMG-CoA (a condensation product of [^13^C_2_]Ac-CoA and [^13^C_4_]acetoacetyl-CoA catalyzed by HMGCS2) over time in cardiac homogenate prepared from pooled NR hearts (Figure 4J). Together, these data indicate that NAD^+^ repletion leads to elevation of SIRT3, ketogenic and ketolytic enzyme levels, acetoacetate generation, and other favorable effects on cardiac energy levels (Figure 4H and 4I). Immunoprecipitation analysis showed a reduction in the acetylation of HMGCS2 in HFpEF myocardium after NR treatment (Figure 4L). These data are consistent with previous reports outlining the deacetylation of HMGCS2 by SIRT3 and, thereby, restoration of ketone body production in the liver.^11^ To investigate metabolic flux in ketogenesis under NR treatment, we examined the impact of NR on the enrichment of HMG-CoA and TCA metabolites using ^13^C_16_-labeled palmitate in isolated primary cardiomyocytes from HFpEF and NR-treated HFpEF hearts. We found a significant increase in labeling from ^13^C_16_-labeled palmitate into the M+4 and M+6 species of HMG-CoA (Figure 4M) in cardiomyocytes from NR-treated HFpEF hearts. We also observed elevated levels of labeled Ac-CoA and TCA intermediate fumarate postincubation with ^13^C_16_-labeled palmitate in these cardiomyocytes (Figure 4M). The levels of α-ketoglutarate, a metabolite that can be derived from either glutamate or isocitrate, did not show significant differences between the 2 conditions (Figure 4M).

### Lack of NAD^+^-Mediated HFpEF Rescue in Hmgcs2 Knockdown Hearts

**Figure 5.**
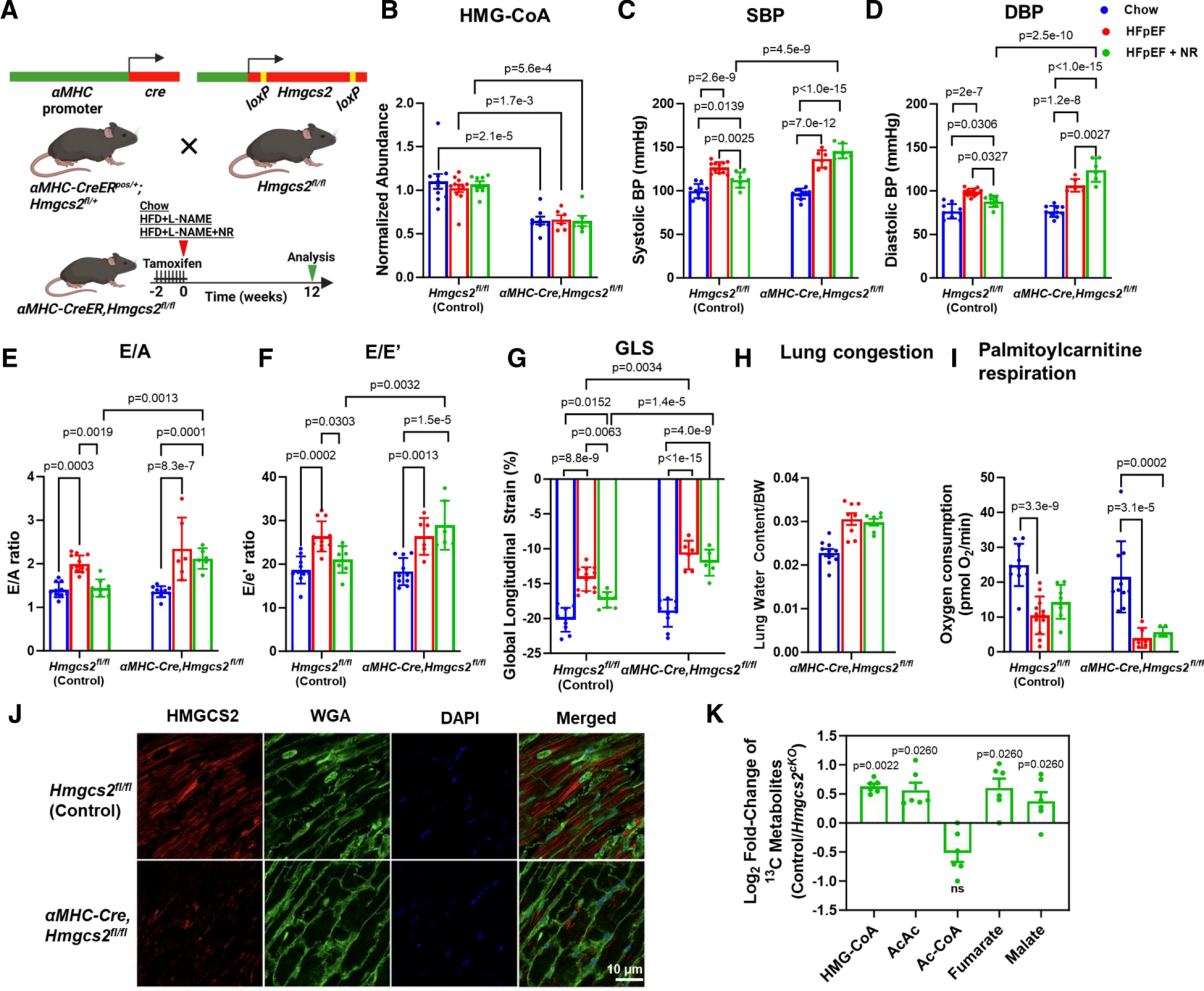
**Cardiomyocyte *Hmgcs2* is critical for the therapeutic effects of NAD+ repletion. A**, Schematic illustration of generation of cardiac-specific Hmgcs2 (3-hydroxy-3-methylglutaryl-coenzyme A synthase 2) knockdown mice. **B**, Metabolite HMG-CoA (β-hydroxy β-methylglutaryl-coenzyme A) levels; (**C**) systolic blood pressure (SBP) and (**D**) diastolic blood pressure (DBP); (**E**) ratio of peak velocity of mitral blood inflow in early diastole to peak velocity of mitral blood inflow in late diastole (E/A); (**F**) ratio between mitral E wave and E’ wave (E/E’); (**G**) global longitudinal strain (GLS); (**H**) lung water content-to-body weight ratio; and (**I**) oxygen consumption rate (OCR) in cardiac tissues utilizing palmitoylcarnitine as a substrate in Hmgcs2 (3-hydroxy-3-methylglutaryl-coenzyme A synthase 2) floxed mice (Hmgcs2^fl/fl^), αMHC (α-myosin heavy chain)-CreER (Cre-loxP recombination) (floxed mice) as controls and αMHC-CreER+, Hmgcs2^fl/fl^, knockdown mice fed by chow, high-fat diet (HFD)+L-N^G^-nitro arginine methyl ester (L-NAME), and HFD+L-NAME+NR for 12 weeks. The sample sizes were n=10/12/8 and n=10/6/6 for the chow/heart failure with preserved ejection fraction (HFpEF)/HFpEF+NR groups, respectively, in both control and *Hmgcs2*-deficient mice. **J**, Representative immunostaining images showing reduced expression of HMGCS2 in the cardiac tissue of *Hmgcs2*-deficient mice compared with control mice. Mice tissues were stained for HMGCS2 (red), wheat germ agglutinin (green), and 4′,6-diamidino-2-phenylindole (DAPI; blue) to delineate cell boundaries and nuclei, respectively (scale bar, 10 µm). **K**, Log_2_-fold change of specific ^13^C-metabolites derived from ^13^C_16_-labeled palmitate in cardiomyocytes isolated from flox-only control mice and HMGCS2-deficient mice; n=6 mice/group. Data are presented as mean±SEM. Statistical significance was assessed using 2-way ANOVA followed by the Tukey post hoc multiple comparisons test (**B**–**I**) and the Mann-Whitney *U* test (**K**). *P*<0.05 was considered significant; exact values are provided in the corresponding figures. CreER indicates Cre-loxP recombination; Cre-LoxP, Cre recombinase–Locus of X-over P1; and Ns, not significant.

To explore the effects of Hmgcs2 deletion on heart function in HFpEF under NR supplementation, we generated cardiomyocyte-specific Hmgcs2-deficient mice using Cre-LoxP–based gene targeting (Figure 5A). Cardiomyocyte-specific excision of Hmgcs2 was induced in adult mice by a single intraperitoneal injection of tamoxifen (50 mg/kg). The real-time quantitative polymerase chain reaction (Figure S5A) and the Western Blot analysis (Figure S5B) were performed on cardiac tissues, confirming a successful reduction in the Hmgcs2 gene and HMGCS2 protein levels following tamoxifen injection. As anticipated, no change in HMGCS2 levels was observed in the liver tissue (Figure S5B). We also observed a decrease in HMG-CoA levels, a direct product of HMGCS2 protein, in murine hearts, confirming the expected decrease in HMGCS2 activity upon gene knockdown (Figure 5B). Lack of HMGCS2 prevented NR (which repletes NAD^+^)-mediated improvements of both systolic and diastolic blood pressures (Figure 5C and 5D), as well as lack of improvement in key indicators of cardiac diastolic function such as the ratio of peak velocity of mitral blood inflow in early diastole to peak velocity of mitral blood inflow in late diastole (Figure 5E), ratio between mitral E wave and E’ wave (Figure 5F), and global longitudinal strain (Figure 5G). We also observed that in the Hmgcs2-deficient mice (αMHC-CreER^+^, Hmgcs2^fl/fl^), there was no improvement in lung congestion, as indicated by the lung water content-to-body weight ratio (Figure 5H). These results highlight the critical role of HMGCS2 in mediating the heart’s adaptive response to NR supplementation and the therapeutic effect of NAD^+^ repletion. To investigate whether fatty acylcarnitine–stimulated respiration through β-oxidation was altered in the HFpEF hearts of either flox-only control (Hmgcs2^fl/fl^) or Hmgcs2 knockdown mice (αMHC-CreER^+^, Hmgcs2^fl/fl^), we measured oxygen consumption rates in fresh tissues using palmitoylcarnitine as substrate with Oroboros oxygraphy. As expected, upon addition of palmitoylcarnitine, the oxygen consumption rate was significantly lower in HFpEF hearts compared with chow control hearts in both flox-only control Hmgcs2^fl/fl^ mice (left red versus blue bars) and cardiomyocyte-specific Hmgcs2 knockdown mice (right red versus blue bars). NR treatment did not restore the oxygen consumption rate induced by palmitoylcarnitine in HFpEF hearts (green bars) to control levels (blue bars); it did normalize the differences seen between HFpEF and chow mice in flox-only control mice (left green versus blue bars) but not in Hmgcs2-deficient mice (right green versus blue bars; Figure 5I). Immunostaining of cardiac tissues showed reduced expression of HMGCS2 in Hmgcs2-deficient mice compared with control mice (Figure 5J). Metabolic flux profiles of control and HMGCS2-deficient hearts were analyzed to determine the role of HMGCS2 in HFpEF under NR supplementation. Results showed a significant increase in ^13^C-label incorporation from ^13^C_16_-labeled palmitate into HMG-CoA and acetoacetate in cardiomyocytes of NR-treated control mice compared with HMGCS2-deficient mice (Figure 5K). In contrast, in the absence of HMGCS2, there was an accumulation of palmitate-derived ^13^C-labeled Ac-CoA in the hearts although this increase was not statistically significant. Furthermore, this Ac-CoA accumulation was associated with a significant reduction in the labeling of TCA cycle intermediates, specifically fumarate and malate, in HMGCS2-deficient cardiomyocytes following incubation with ^13^C_16_-labeled palmitate (Figure 5K).

Additional analysis presented in Figure S6A showed that palmitoylcarnitine-mediated respiration was significantly lower in the hearts of the NR-treated Hmgcs2-deficient HFpEF mice compared with NR-treated flox-only control mice. This reduction in palmitoylcarnitine-mediated respiration, alongside a significant decrease in cardiac HMG-CoA levels, as shown in Figure S6B, suggests that the absence of HMGCS2 impairs the conversion of FA-derived Ac-CoA into HMG-CoA. In addition, our findings indicate that NR-treated Hmgcs2-deficient HFpEF mice exhibit lower ATP/ADP and phosphocreatine/ATP ratios, suggesting compromised energy status and impaired mitochondrial function (Figure S6C).

We also expanded our investigation to include the cardiac lipidome, as presented in Figure S6D and S6E, and our results indicated that the downregulation of FAO in Hmgcs2-deficient mice receiving NR supplementation led to the accumulation of acylcarnitines and triacylglycerols compared with the NR-treated flox-only control mice. Specifically, we observed a significant increase in long-chain to very-long-chain acylcarnitines (C18–C26) and the accumulation of 70 distinct triacylglycerol species in the hearts of these mice. Further analysis revealed that these triacylglycerols are predominantly composed of long-chain FAs with 14 to 18 carbons and lower double bond content (<3), including FAs such as myristic (14:0), palmitic (16:0), palmitoleic (16:1), stearic (18:0), and oleic (18:1), as highlighted in Figure S6F. In contrast, NR-treated flox-only control mice did not show accumulation of triacylglycerols, particularly of those with FA chains between 14 and 18 carbons and low double bonds, suggesting the requirement of HMGCS2 in the restored normal lipid metabolism upon supplementation with NR, thereby maintaining normal cardiac lipid profiles in these mice.

## Discussion

It was reported several decades ago that it is the depletion of oxaloacetate, after excess lipid accumulation in the liver, which serves as the key ketogenic stimulus.^36^ Ac-CoA produced from FAO would normally combine with oxaloacetate to form citrate and enter the TCA cycle; however, because oxaloacetate is depleted and the rate of production of Ac-CoA exceeds the capacity of citrate synthesis, Ac-CoA is instead used to form ketone bodies.^37^ Remarkably, we see the exact same conditions in HFpEF myocardium (Figure 3B and 3C), reaffirming the same canonical generative pathway in HFpEF hearts in this murine model. Reduced myocardial phosphocreatine/ATP ratio suggests a state of energy depletion in the heart,^38^ augmenting the stimulus to seek other sources of energy, and ketones are a preferred alternative energy source of the heart.^6^ The current paradigm suggests that robust HMGCS2 expression only occurs in hepatocytes and gut epithelial cells: extrahepatic tissues do not contribute ketone bodies to the circulation, and they internalize ketones down a concentration gradient via MCT1/2-dependent mechanisms.^37,39^ The myocardium is the highest ketone body consumer per unit mass, and cardiomyocytes oxidize ketone bodies in direct proportion to their delivery,^40–43^ especially during carbohydrate-restricted states. The balance of ketone body production and disposal determines the steady-state circulating concentrations of ketone bodies.^37^ However, we found that cardiac expression of the main βHB transporter, MCT1, was decreased in HFpEF hearts, while the levels of the rate-limiting ketogenic enzyme were dramatically upregulated in HFpEF hearts. Our study shows that metabolic stress in the HFpEF heart compromises its ability to use glucose as a fuel source due to insulin resistance. This removes a crucial fuel source, leading to significant energy deficiency. In response to the energy shortfall, the HFpEF heart actively increases its uptake of FAs and its enzymatic capacity for FAO.^44^ This is evident in our study, where we demonstrated an increase in the expression of enzymes involved in FA transport and oxidation at the protein level. Despite an increase in FAO enzymes in the hearts, we observed a decrease in FAO activity and an accumulation of long-chain to very-long-chain acylcarnitines and triacylglycerols, indicating incomplete FAO.^45^ The accumulation of triacylglycerols, primarily composed of saturated or monounsaturated long- to very-long-chain FAs, suggests that these FAs remain unmetabolized and are subsequently re-esterified into triacylglycerols. In a study by Tong et al,^13^ it was shown that hyperacetylation of FAO enzymes, such as VLCAD and HADHA (trifunctional enzyme subunit α), contributes to FAO impairment in HFpEF hearts. This acetylation modification, potentially mediated by Ac-CoA, is evident in the hyperacetylation of VLCAD and HADHA. Recent evidence,^46,47^ including a study by Pougovkina et al,^48^ suggests that Ac-CoA generated from FAO can lead to protein acetylation modifications. Upregulation of HMGCS2 could potentially serve as a compensatory mechanism to remove excess Ac-CoA and enable FAO products to be utilized in ketolysis. This is supported by our findings where we did observe improved FAO after NR treatment in wild-type hearts where HMGCS2 was present but did not observe an improvement in NR-mediated FAO in the absence of HMGCS2. Together, this suggests that HMGCS2 plays a crucial role in NR-enhanced FAO in HFpEF.

In addition, HFpEF murine myocardium showed a decreased expression of key ketolytic enzymes BDH1 and SCOT/OXCT1, accompanied by increased myocardial βHB-to-acetoacetate ratio, suggesting an inability of the HFpEF heart to use βHB for defense of metabolic stress. Mitochondrial conversion of βHB to acetoacetate is dependent on BDH1 activity and is directly proportional to the NAD^+^/NADH ratio, which is critical for maintaining myocardial energy homeostasis.^37^ Our HFpEF hearts had decreased levels of BDH1, NAD^+^/NADH ratio, and acetoacetate but increased levels of βHB and FAO proteins, suggesting an immediate stimulus to generate sufficient acetoacetate through endogenous ketogenesis. However, we are not proposing that cardiac ketogenesis is important outside the heart or contributes to circulating ketones. Rather, the data presented in this study reveal an acute sensitivity of the canonical ketogenic enzyme in the heart to the metabolic inflexibility and energetic demands seen in HFpEF.

It has been suggested that apparent extrahepatic ketogenesis can occur in the context of decreased ketone oxidation/consumption,^37^ whereby decreased ketone oxidation leads to ketone body accumulation and gives the appearance of de novo ketogenesis. Non-HMGCS2 pseudoketogenesis has been described in extrahepatic tissues and occurs due to the reversible enzymatic activity of ACAT (acetoacetyl-CoA thiolase) and CoA transferase (OXCT1).^49,50^ While this remains a possible contribution to the ^13^C enrichment of acetoacetate that we saw in the human and murine myocardium, our data confirm canonical HMGCS2 ketogenesis through time-dependent ^13^C enrichment of HMG-CoA (the key irreversible step in ketogenesis), which would not occur via reverse thiolase or CoA transferase.

Previous studies of rodent type 1 diabetes models reported upregulation of myocardial mRNA levels of HMGCS2 in mice^51^ and both its mRNA and protein levels in rats.^52^ However, these studies did not determine enzymatic activity, posttranslational modification, or the relative changes in FAO, glucose metabolism, or citric acid cycle changes at the metabolite level, essential to pinpoint the transition to ketogenesis. In fact, our comprehensive assessment of HMGCS2-mediated ketogenesis is instructive. Measuring only the mRNA or protein levels would have provided a profoundly misleading conclusion. Shimazu et al^11^ revealed that the activity of liver HMGCS2 is dependent on continual deacetylation on site-specific lysine residues (Lys310, Lys447, and Lys 473) by SIRT3. While our data suggest that this is also a potential mode of regulation in the heart, further work is required to identify the site-specific lysine residues in myocardial HMGCS2. SIRT3 is the major deacetylating enzyme, itself dependent on the NAD^+^/NADH ratio.^35,53^ In addition to its role in deacetylating HMGCS2, there are other roles of SIRT3, which are important to consider in the context of heart failure and HFpEF, including maintaining mitochondrial function^54–56^ and preventing cardiac hypertrophy.^54^

We next explored the mechanistic role of HMGCS2 in HFpEF and whether it is required for NAD^+^-mediated rescue. To this end, we generated conditional, cardiomyocyte-specific Hmgcs2-deficient mice. The lack of cardiac improvement imbued by NR supplementation with Hmgcs2 knockdown highlights the enzyme’s importance in maintaining cardiac metabolic homeostasis, especially as it pertains to NAD^+^-dependent mechanisms, which may involve its regulation of lipid metabolism and energy production as indicated by disrupted FA utilization and diminished energy status.

Remarkably, the ketone changes in murine HFpEF were consistent with human HFpEF. Murine HFpEF myocardial βHB levels were nonsignificantly increased, and acetoacetate levels significantly decreased. Human HFpEF hearts had a significantly increased uptake of acetoacetate but not βHB. The selective uptake of acetoacetate occurs along a gradient of myocardial acetoacetate deficiency via MCT1/2 transporters, which likely results from increased myocardial AcAC consumption in HFpEF. Our patients with HFpEF were defined by elevated pulmonary capillary wedge pressure during exercise (≥25 mm Hg), a more stringent parameter to confirm HFpEF. Exercise pulmonary capillary wedge pressure has been shown to be a more accurate diagnostic criterion for HFpEF, differentiating HFpEF from non-HFpEF disease states.^14,15^ Using transcardiac gradients, others have shown increased ketone contribution of total ΔO_2_ in HFpEF compared with HFrEF^57^ and increased ketone body uptake in patients with HFrEF and aortic stenosis compared with controls.^58^ However, to our knowledge, we are the first to demonstrate increased cardiac uptake of acetoacetate in human HFpEF hearts compared with HFrEF hearts.

Much recent work has focused on the salutary effects of ketone bodies in heart failure.^6–9,59^ Exogenous ketone bodies are cardioprotective in HFpEF, allowing the heart to extricate energy at a lower oxygen cost^60^ and suppressing myocardial inflammation.^9^ Indeed, we show human HFpEF hearts extricate more acetoacetate than HFrEF hearts. However, augmenting the local generation of ketones by the heart would serve as a valuable therapeutic target for multiple cardiac diseases. Herein, we show that the heart is not just a consumer of ketones provided by the liver. We definitively demonstrate that human hearts are capable of ketogenesis via the canonical ketogenic enzyme, with a dramatic, but dysfunctional, upregulation of the ketogenic enzyme under the confluence of stresses seen in HFpEF, in an apparent strategy to eliminate metabolic blockade and rescue energy deprivation.

Crucially, we demonstrate that myocardial ketogenesis can be augmented with the repletion of cofactor NAD^+^ that restores SIRT3 protein levels and deacetylase activity to maintain the function of HMGCS2. As noted above, previous research has shown that deacetylation of HMGCS2 by SIRT3 at specific lysine residues restores HMGCS2 function and increases the production of ketone bodies in the liver.^11^ Future studies are required to determine the biological relevance of specific lysine deacetylation sites critical for HMGCS2 activation in HFpEF hearts.

In conclusion, given the multifold benefits of ketones in HFpEF including provision of energy and suppression of inflammation,^9^ our study adds an important new aspect demonstrating a mechanistic role of the ketogenic system in the heart itself. We think that these results reframe our understanding of cardiac ketone metabolism, highlight the mechanistic role of the myocardial ketogenic pathway in HFpEF, and suggest that this pathway is a potential therapeutic target in HFpEF.

### Limitations

Given the heterogeneity of HFpEF, our murine model likely represents a specific, if the most common, subset. However, it is unclear whether our findings are relevant to other causes of HFpEF, for example, infiltrative cardiomyopathy.

Our data demonstrate the rescue of diastolic dysfunction and increased expression of ketone body oxidation enzymes in HFpEF mice following NR supplementation, with upregulation of both ketogenic and ketolytic capacity upon NAD^+^ replenishment. However, we did not quantify the contribution of ketone oxidation to the increase in myocardial ATP levels in the NR-treated mice.

It is important to note that we did see upregulation of HMGCS2 staining in hypertrophic cardiomyopathy, which presents with diastolic heart failure in the absence of metabolic duress and, perhaps, suggests a more pervasive role of ketone metabolism in hypertrophy/diastolic impairment.

We acknowledge that our FAO analysis has been confined to medium-to-long-chain saturated FAs for FAO activity assessment, which limits our scope to this specific class and has not examined the broader variety of FAs, especially those differing in chain lengths and saturation. However, we did validate our FAO oxidation assays using 2 independent methods. Future work will include the diverse types of FAs for a more comprehensive understanding of FAO processes in HFpEF.

## ARTICLE INFORMATION

### Acknowledgments

The authors thank SydneyMS for their technical and instrumentation support in our study. The authors also thank Sydney Imaging’s Preclinical Imaging Facility for their assistance and access to cardiac imaging instruments. The authors acknowledge the technical assistance of Sydney Microscopy & Microanalysis, The University of Sydney Node of Microscopy Australia. The authors also thank The Yilmaz Laboratory at the Massachusetts Institute of Technology for their crucial assistance in providing the *Hmgcs2*^*fl/fl*^ mice, which were developed in their laboratory.

### Author Contributions

Y.C. Koay and J.F. O’Sullivan conceived the study and designed the experiments. Y.C. Koay, B. McIntosh, Y. Cao, Y.H. Ng, Y. Han, S. Tomita, A.Y. Bai, B. Hunter, Y. Cao, and M. Larance performed the experiments. Y.C. Koay, B. McIntosh, S. Tomita, B. Hunter, M. Larance, and J.F. O'Sullivan analyzed data. A. Misra conceptualized and oversaw the generation of genetically modified mice. D.M. Kaye performed and analyzed the Alfred Hospital HFpEF/HFrEF study (Heart Failure With Preserved Ejection Fraction/Heart Failure With Reduced Ejection Fraction). Y.C. Koay, J.F. O’Sullivan, A.J. Lusis, and S. Lal reviewed the data. Y.C. Koay and J.F. O’Sullivan wrote the article, which was reviewed by all authors.

### Sources of Funding

Y.C. Koay is supported by a National Heart Foundation Future Leader Fellowship (Level 1; grant NHF107180) and an New South Wales (NSW) Cardiovascular Collaborative Grant (OHMR23-251985). J.F. O’Sullivan was supported by the NSW Health Early Mid Career Fellowship and Clinician-Scientist Awards (DOH1003 and DOH1006), the National Heart Foundation Future Leader Fellowship (grant NHF104853), and the National Health and Medical Research Council-Medical Research Futures Fund (NHMRC-MRFF) Cardiovascular Health Mission (grant 107180). C.M. Loughrey is funded by the British Heart Foundation Program grant (RG/20/6/35095).

### Disclosures

None.

### Supplemental Material

Supplemental Materials and Methods

Table S1

Figures S1–S6

Major Resources Table

References 61–77
